# Drought-induced shift in tree response to climate in floodplain forests of Southeastern Europe

**DOI:** 10.1038/s41598-018-34875-w

**Published:** 2018-11-07

**Authors:** Stjepan Mikac, Anja Žmegač, Domagoj Trlin, Vinko Paulić, Milan Oršanić, Igor Anić

**Affiliations:** 1 University of Zagreb, Faculty of Forestry, Department of Forest Ecology and Silviculture, Svetošimunska 25, 10002 Zagreb, Croatia; 2 University of Zagreb, Faculty of Forestry, Croatian Dendroecology Laboratory, Svetošimunska 25, 10002 Zagreb, Croatia; 3 Croatian Academy of Sciences and Arts, Zrinski trg 11, 10000 Zagreb, Croatia

## Abstract

Floodplain forests are the most rapidly disappearing ecosystem in the world, especially in temperate regions of Europe where anthropogenic influence has been pronounced throughout history. Research on primeval forests is crucial to further our understanding of their natural dynamics and interaction with climate but is limited by the lack of such preserved forests. The aim of this study was to investigate how a primeval floodplain forest in Southeastern Europe has responded to climate variability during the last 250 years through comparison of tree growth and climate, canopy disturbance and recruitment dynamic of two dominant tree species with different tolerances to flooding/drought. Our analysis revealed induced stress caused by several consecutive severe drought events in the 1940s, which led to a significant increase in sensitivity to increasing temperatures and decreasing river water levels. This trend is particularly pronounced in pedunculate oak. Age structure analysis revealed one larger episode of oak regeneration culminating after periods of intense growth release. Such period co-occurs with summer drought, which is part of a complex system of natural disturbances and a significant natural driver of the cyclical regeneration of primeval oak ecosystems.

## Introduction

Global forest decline caused by drought has been recorded worldwide and has significantly increased since 1970^1,2^. Recent changes in climate are associated with increased temperatures and changes in precipitation patterns, with more frequent, prolonged and intense episodes of drought as a consequence. Such events result in long-lasting changes in ecosystem function, community composition and structure, especially in water sensitive ecosystems such as floodplain forests^3^. Lowland floodplain forest ecosystems are characterized by high productivity, diverse microhabitat conditions and considerable biodiversity^4^. They are widespread in all biogeographic regions of the world on alluvial deposits of large rivers with which they have a constant hydrologic interaction^5^. According to a study by Tockner *et al*. in 2002^6^, the world’s remaining floodplain forests cover an area of approximately 2.24 × 10^6^ km^2^.

The continuous expansion of settlements and infrastructure, as well as exploitation of natural resources, has ultimately resulted in the widespread disappearance of primeval lowland floodplain ecosystems^7,8^. In Europe, natural lowland floodplain forests have all but vanished, and with them, a very important research reference point for forestry and ecology. In the last century deforestation due to agriculture has wiped out 90% of Europe’s floodplain forests^9,10^. The remnants of relatively natural forest occur mostly in Eastern and Southeastern Europe^3,11^. Apart from deforestation, floodplain forests have been impacted by numerous activities, particularly river regulation (construction of dams, dykes, drainage systems, etc.). These interventions have disrupted the sensitive flood patterns and assisted the progression of mesohydric species^12–14^. Regional episode of pedunculate oak (*Quercus robur* L.) decline were recorded during the 20th century in floodplain forests in almost all of Europe^15^. As oak and other species die out, another problem in lowland floodplain ecosystems is the spread of mesohydric species, such as hornbeam (*Carpinus betulus* L.), which are becoming increasingly dominant, especially in drier, oak dominated habitats. In the last 20 years significant decline in narrow-leaved ash (*Fraxinus angustifolia* Vahl) was observed through the whole landscape. The greatest threat to the stability of forest ecosystems of narrow-leaved ash is currently posed by the phytopathogen *Hymenoscyphus fraxineus* T. Kowalski^16^ but also constant increase of temperature and environment dryness. The continuation of oak and ash decline could have long-lasting consequences for biodiversity as well as for the European forestry sector and is a huge challenge for nature conservation. Forest management in Europe strives to implement a close-to-nature approach based on mimicking natural stand dynamics^17^. In order to better understand the dynamics of natural lowland floodplain forests and their interaction with climate, primeval natural ecosystems need to be studied. However, such studies are lacking in Europe since it is almost impossible to find a primeval lowland floodplain area with an exclusively natural composition and structure. Therefore, we carried out this study in an area with one of the best preserved floodplain forest complex of pedunculate oak and narrow-leaved ash in Europe – Lonjsko Polje Nature park (LPNP)^13^.

The aims of this study were to: (1) analyze the long-term growth sensitivity of oak and ash to climate variability and changes in river water regime; (2) determine if climate is the driver of growth releases in the study area; (3) reconstruct disturbance history through analysis of the relationship between canopy disturbance, tree growth and recruitment.

We expect that: (1) oak growing on drier sites is more sensitive to precipitation, PDSI and river water regime than ash; (2) extreme climatic events drive the growth releases and the establishment of recruitment in these forests.

## Materials and Methods

### Research area

The floodplain forests in the lowland part of Croatia occupy an area of about 1,980 km^2^, mainly along the Sava, Drava and Danube rivers. One of the best preserved natural floodplain regions in Europe is the Sava River basin, with an area of 97,713 km^2^. Geographically, it lies between 13.67E–20.58E longitude and 42.43N–46.52N latitude. It represents 12% of the Danube River basin, making it the second largest Danube sub-basin. Its preserved naturalness is the result of historical circumstances. Up to the 19th century the Sava River was a natural barrier between the Austro-Hungarian and the Ottoman empires. In that period, the forests were under strict control of the military authorities and were protected. The largest and best preserved floodplains and forest ecosystems in Europe can be found in the central part of the Sava River basin and make up what is called “The Blue Heart of Europe” (Lonjsko polje Nature Park, LPNP). The area of approximately 511 km^2^ hosts a mosaic of preserved wild lowland floodplain forests and alluvial swamps. The dominant tree species are narrow-leaved ash (*Fraxinus angustifolia* Vahl) and pedunculate oak (*Quercus robur* L.). Ash is found on wet and heavy gley soil and forms the swamp (“wet”) forest edge which is regularly flooded during the year (spring & autumn), while oak is found on drier terrain, often out of the reach of regular annual floods, and it represents the upper (“dry”) edge of these forests. Oak is the main indicator of mesophilic conditions, but common hornbeam (*Carpinus betulus* L.) also occurs and is becoming progressively more dominant in floodplain ecosystems. This kind of mosaic alternates throughout the entire area regardless of the distance from the riverbed due to the specific micro-terrain (micro-rises and micro-depressions) caused by the settling of alluvial deposits.

**Figure 1:**
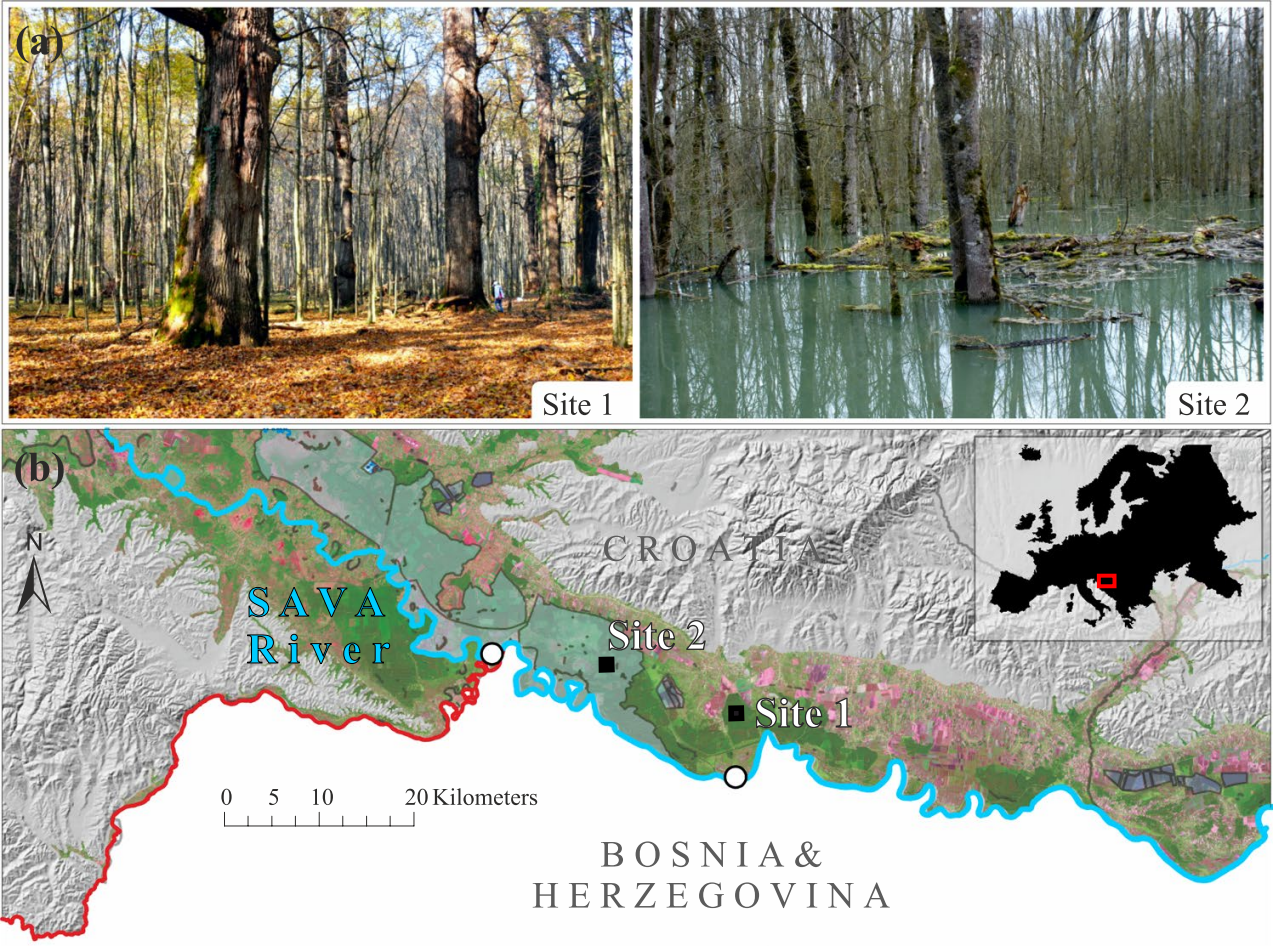
Location of the study sites. Photographs of oak (dry, Site 1) and ash (wet, Site 2) sampled stands (**a**). Positions of sampling areas (black square), the location of the hydrologic water level monitoring station (white dots) and natural, regularly flooded area (shaded polygon) (**b**).

This study was conducted on the eastern edges of the LPNP area, which is under the direct influence of flooding from the Sava River and its tributaries. The area is characterized by a humid continental climate, with an average annual air temperature of 9.5 °C and total precipitation of 870 mm with a maximum in June and a minimum in February. Evaporation is estimated in the range of 520–600 mm per year. For the study, two areas (Fig. 1a,b) were chosen as representative of extremely wet habitats (swamp edge) and extremely dry habitats. The areas were required to be (i) absent of direct anthropogenic influence (cutting, grazing etc.), (ii) representative of marginal populations with respect to the wetness of the habitat, (iii) exposed to the same climate conditions (precipitation and temperature) and (iv) similarly distant from the Sava riverbed. The first area is a mixed stand of pedunculate oak and common hornbeam in the Prašnik Forest Reserve (dry site, elevation = 95 m, distance to river = 3,2 km) and the second is a pure narrow-leaved ash stand that forms the border between the forest and permanent wetlands (wet site, elevation = 90.5 m, distance to river = 2,9 km). Prašnik, with an area of 50 ha, is the last representative example of the primeval lowland forests that encompassed approximately 400 km^2^ of the research area before World War I. The wood volume is approximately 550 m^3^ ha^−1^ with 24 trees ha^−1^. Pedunculate oak trees reach impressive dimensions of up to 260 cm in diameter and 45 m in height. A dramatic change in species composition has been observed in the last five decades, with common hornbeam having progressed to the point where it is currently dominating the understory. The second site consists of pure stands of narrow-leaved ash that grow on the boundaries of constantly wet alluvial swamps. The wood volume is approximately 320 m^3^ ha^−1^ with 160 trees ha^−1^. These narrow-leaved ash stands most probably arose by natural succession during the last 200 years. This was inferred by studying historical maps from the late 18th century (http://mapire.eu/en/).

### Field sampling

In Prašnik (dry site) we established a grid of 10 circular experimental plots, each amounting to 2500 m^2^ that were evenly spread through the whole reserve area. Within each plot we positioned and sampled all trees and dead wood with a diameter over 10 cm (Supplementary Fig. 1**)**. At the swamp boundary (wet site) we established three experimental plots, each amounting to 800 m^2^, where all of the ash trees were sampled. Two cores per tree were collected with a Pressler borer at approximately 1.30 m above ground level^18^. After collection, preparation and drying of the samples, we carried out standard coarse and fine sample processing, incrementally increasing the sandpaper granulation (granulation of 120 to 600).

### Climate data

**Figure 2:**
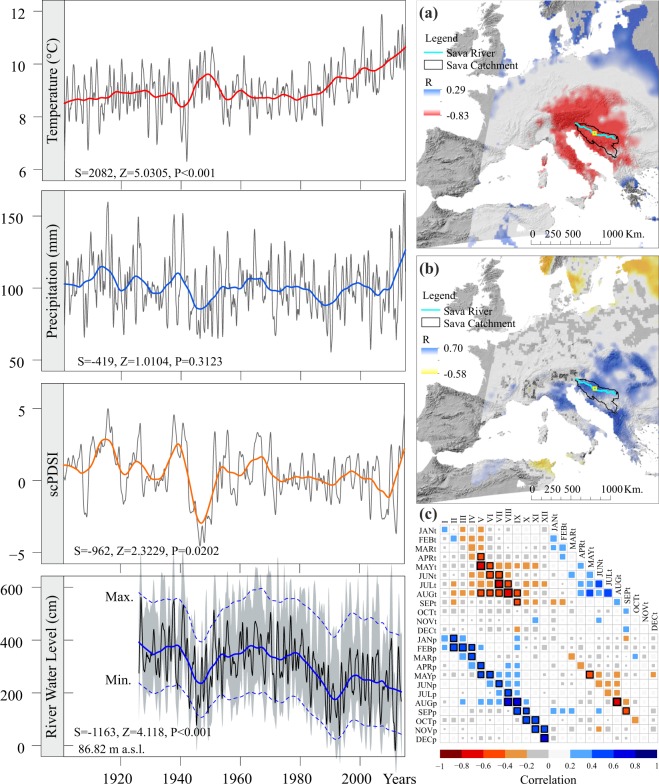
Long-term trend of mean annual climate data (Temperature, Precipitation, scPDSI) and River Water Level. Spatial field correlation between the mean 12-monthly Sava River Water level with E-OBS 14.0 current year Tmax (averaged May–August) (**a**) and gridded precipitation (**b**) for the period 1950–2014. The correlation matrix between average monthly water levels (I–XII), temperature (t) and precipitation (p) for the period 1926–2014 (**c**).

Climate data (mean monthly air temperature, precipitation and standardized Palmer drought index – scPDSI) were obtained from the gridded CRU TS3.24.01 database (Fig. 2) with a spatial resolution of 0.5° × 0.5° for the 1901–2015 period using the KNMI Climate Explorer platform^19^ (http://climexp.knmi.nl). For the long-term climate correlations (>100 years), analysis was done using data (mean monthly temperature and monthly precipitation sums) from the HISTALP database (http://www.zamg.ac.at/histalp/). The database contains monthly homogenized precipitation data from 192 weather stations and homogenized air temperature data from 131 weather stations in the broader Alps and Dinarides area (4° to 19°E latitude and 43° to 46°N longitude)^20^. We used grid-mode-2 series that represent absolute monthly air temperature and precipitation values in a 5 × 5 minute resolution (4° to 19°E latitude and 43° to 46°N longitude). Hydrological parameters (mean monthly water level and river discharge values) for the Sava River in the 1926–2014 period were obtained from the Croatian Meteorological and Hydrological Service (http://meteo.hr/). The distance between dry site, wet site and nearest river gauges were 12 km and 7 km, respectively.

### Chronology development

Tree-ring width was measured with a LINTAB measuring table with 0.01 mm precision, equipped with OLYMPUS binoculars and a polarized light source. Cross-dating of samples was done both visually and using the TSAP-Win™ dendrochronological software (http://www.rinntech.de). Cross-dating quality was verified using COFECHA program^21,22^ by checking the consistency of ring width series among trees from the same site. We averaged two cores for each tree, thereby obtaining one representative series per tree (Supplementary Fig. 2**)**. For each core cambial age was estimated using concentric circles method. Pith estimates of 10 missing rings or more were not included in the age data set.

**Figure 3:**
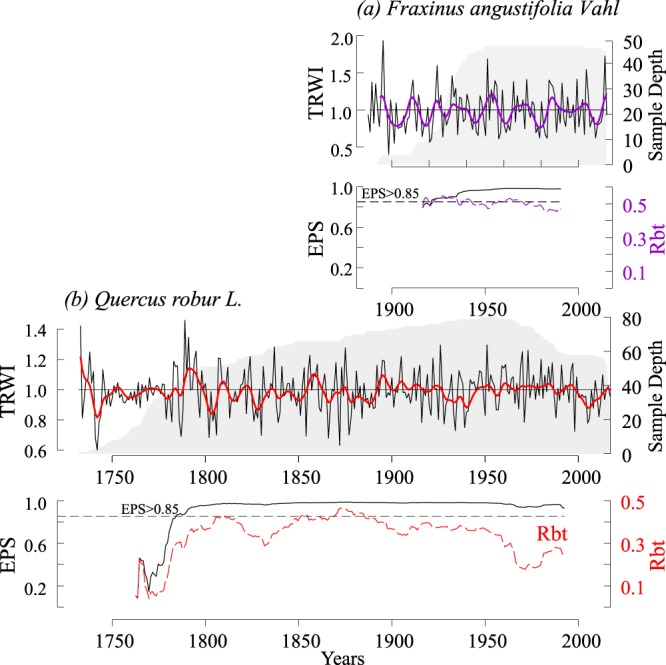
Residual tree-ring index chronologies smoothed with 10 years low pass filter to highlight decadal high-frequency variability (violet and red). Running EPS and running Rbt statistics (Inter trees correlation) for *Fraxinus angustifolia* (**a**) and *Quercus robur* (**b**). EPS and Rbt was calculated using a 50-year moving window.

Detrending, that is, removing frequency variability as a consequence of the biological age effect as well as standardization was done on individual tree-ring width series using the “dplR” package in R^23^. Several methods (Negative exponential curve, Regional curve standardization - RCS, Signal-Free RCS - RCSsf, C-method, Spline) were used (Supplementary Fig. 3**)**. Following standardization, individual series were calculated using Tukey’s biweight robust mean^24^ to obtain a residual chronology^25^ (Tree-ring width index - TRWI) which was used in all subsequent analyses (Fig. 3). Since the correlation to climate of chronologies obtained through the mentioned methods showed almost no difference (Supplementary Fig. 4), the Spline method (frequency response of 0.50 cut off at 0.67 series length) was chosen.

The quality of the obtained chronology was assessed by using several dendrochronological statistics: mean sensitivity (*MS*), which is a measure of year-to-year variability in the tree growth series^26^ calculated as the difference between each two successive rings divided by their mean^27^; first-order autocorrelation of raw data (*AC1*), which determines the variance of the current year’s growth that is explained by the previous year’s growth^28^; the expressed population signal (EPS), used to assess chronology reliability where EPS value over 0.85 quantifies the degree to which the constructed chronology represent the hypothetical population^29^ and mean interseries correlation (*Rbar*) (Supplementary Table. 1).

### Climate-growth analysis

Climate–growth relationships were assessed by correlation function as well as response function analysis. In correlation functions, the coefficients calculated between the tree-ring chronology and monthly climatic variables are univariate estimates of Pearson’s product moment correlation. In response functions, the coefficients are obtained through multiple regression using the principal components of monthly climatic data to estimate ring-width growth indices. They are interpreted as average effect of the fluctuation of that monthly climatic variables on tree growth. This regression model is used in tree-ring studies to identify the climate origin of variability in the chronologies through avoidance of intercorrelations between climatic predictors^26^.

**Figure 4:**
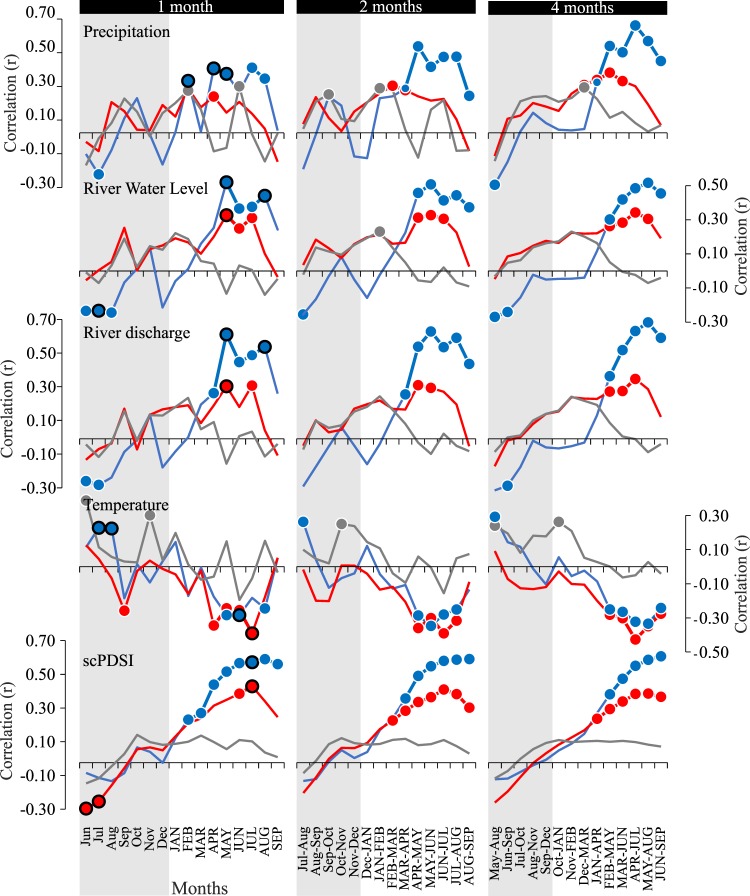
Bootstrapped mean monthly and seasonal correlation between TRWI and climate. *F*. *angustifolia* (blue) and *Q*. *robur* (old – red, young - gray) for selected climate factors (Temperature, Precipitation and scPDSI) and hydrological parameters (Sava River water level and river discharge) for the 1950–2015 period. Statistically significant values are marked with a dot (*p*-value < 0.05) and significant response coefficient with a black bordered dot. Shaded area highlights the correlation values with months of previous year.

Analysis was performed with the “*treeclim*” package in R^30^ for a period of 16 months (June of the previous year to September of the current year) between climate and hydrologic data and residual chronology (Fig. 4). The significance (*p*-value < 0.05) of each coefficient was evaluated using 1000 bootstrap replications mimicking DENDROCLIM2002 software^31^. Analysis was performed for the period of 1950–2014 (determined by the greatest frequency and quality of meteorological data). Seasonal correlation analysis was done using “*treeclim*” package^30^ for a 95% significance level for different season durations from 2 to 6 months in monthly increments. In the results we show seasonal durations for 2 and 4 months (Fig. 4).

### Temporal stability of Climate-growth relations

**Figure 5:**
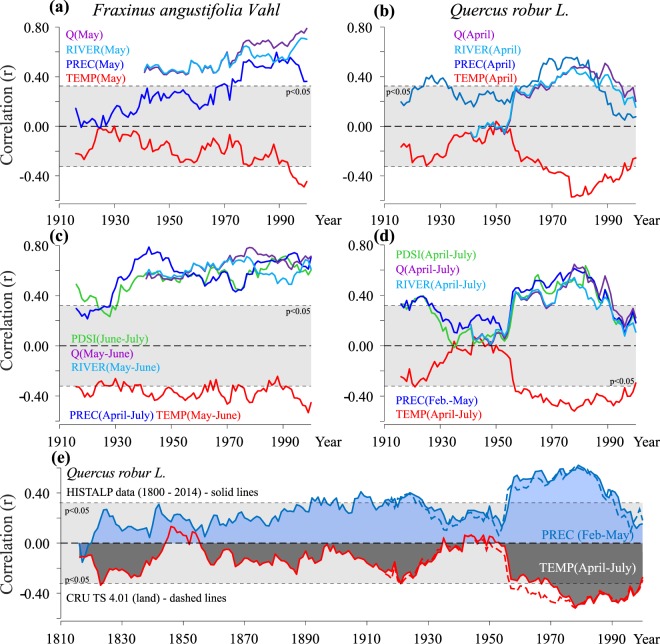
Moving correlation between TRWI and most significant monthly (**a**,**b**) and average seasonal climate factors from CRU TS4. 01 and HISTALP and hydrological data (river water level - RIVER, and river discharge - Q) (**c**,**d**) using 30 years moving window for *F*. *angustifolia* and *Q*. *robur* for the period 1901–2014 and longer (1801–2014) period (**e**).

Temporal stability of the climate signal was analyzed using moving window correlations with a 30-year interval (Fig. 5). Analysis was performed with the most significant monthly and seasonal variables for the 1901–2014 period using CRU TS3.24.01 climate data, with additional analysis of pedunculate oak correlations for a longer period (1800–2014) using HISTALP climate data (Fig. 5e).

### Disturbance analysis

**Figure 6:**
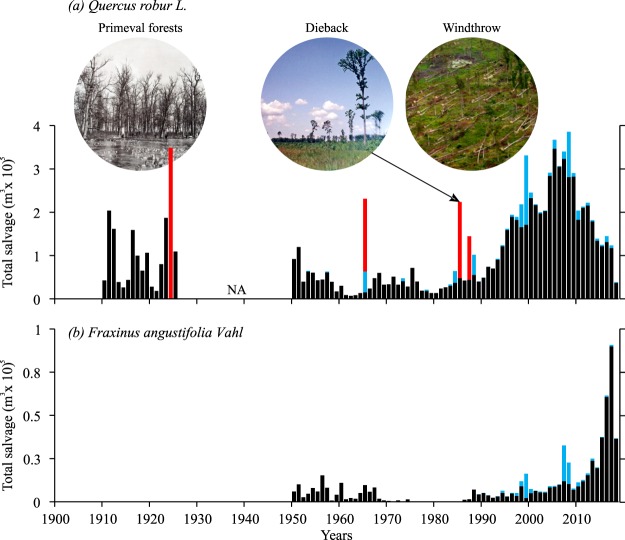
Salvage logging data for the lowland area of Croatia. Black histograms represent sum of drought induced annual salvage logging of oak (**a**) and ash (**b**) and windthrow (blue) with individual high-severity disturbance events (red). NA- data not available for this period.

We collected documented and archived records of salvage logging (drought induced) and disturbances for the entire lowland region in Croatia for the past century. We extracted information on the agent of past disturbances and quantity of salvaged wood (m^3^ ha^−1^) (Fig. 6).

This data provides information from a large area giving us a useful insight into past disturbance regimes and general trends in decline, but it should be kept in mind that it is obtained from managed and seminatural forest which can differ in their resistance to disturbances^32^.

**Figure 7:**
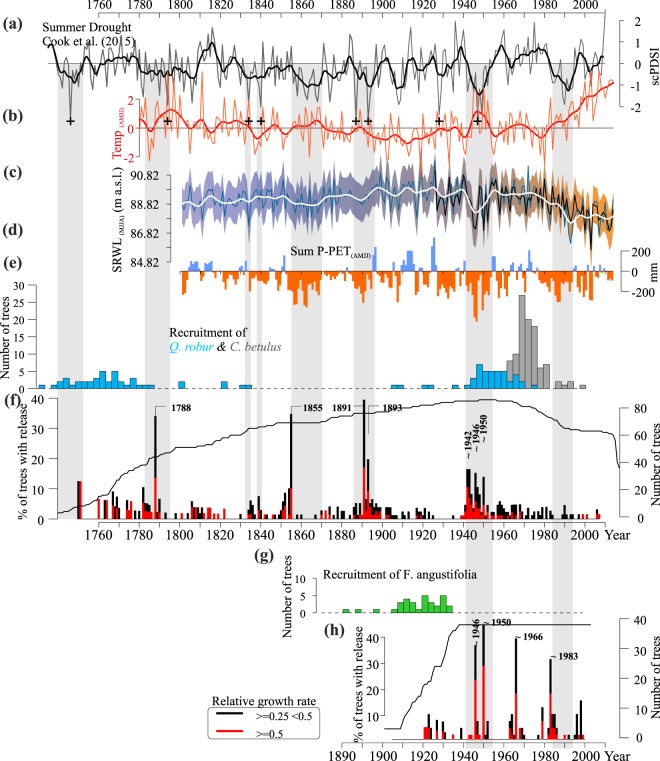
Disturbances chronology and climate variability. PDSI reconstruction for summer (June–August) droughts severity (Cook *et al*., 2015) (**a**). Mean temperature difference from April to July (**b**, most severe droughts marked with plus signs). Mean Sava river water level from May to August (**c**). Difference in precipitation and potential evapotranspiration (PET) for the period (April–July) calculated using long-term climate data series received from the HISTALP database for sum for the last two years (**d**). Recruitment of oak and hornbeam (**e**) and ash (**g**) based on cambial age. Disturbance chronology with moderate (black) and major releases (red bar) displayed in annual interval for pedunculate oak (**f**) and narrow-leaved ash (**h**).

For disturbance analysis in our study, series from trees older than 150 years were used (80 trees). For each tree, the percentage change in growth was calculated according to the method proposed by Nowacki and Abrams^33^. This method uses a percent growth change equation:

$$ \% {\rm{GC}}=(({\rm{M2}}-{\rm{M}}1)/{\rm{M}}1))\ast 100,$$

where % GC is percent growth change from preceding to superseding 10-yr radial average, M1 is the preceding 10-yr mean radial growth (exclusive of the current year) and M2 is the superseding 10-yr mean radial growth (inclusive of the current year). The minimum threshold for release is 25% growth change for moderate and >50% for major release. The percentage of trees showing releases was plotted against time (Fig. 7).

## Results

### River water level

The analysis of monthly water level data showed a significant decrease in the Sava River water level from 1926 to 2014, with the decrease being especially pronounced after 1980 (Fig. 2). This is caused mostly by recent increases in temperature, especially in the summer period from May to August (*r* = −0.79, *p*-value < 0.001). Individual monthly and average seasonal air temperature and river water level correlations in July and August exhibit the largest values (R = −0.72, *p*-value < 0.001) (Fig. 2.). Total annual precipitation for the LPNP area is 870 mm. Of this amount, 450 mm occurs in the vegetation period (May to September), nearly the total amount of actual evapotranspiration. The total variability of the water level explained through the negative influence of summer air temperatures (May–August) and the positive influence of precipitation (April–August) is 68% (*p*-value < 0.001).

### Tree-ring statistics

For the climate-growth analysis a total of 215 samples of pedunculate oak and 85 samples of narrow-leaved ash were dated. Since some of the oak samples were in very poor condition upon extraction, with visible rot and variable wood consistency, only 208 samples of oak were used for the climate-growth analysis (Supplementary Fig. 2d). In some cases, the oak tree dimensions exceed 2 m in diameter, making sampling particularly difficult. Bearing in mind that this area is protected, we limited the size of the sample to the smallest possible. The chronology range is 1732–2017 for oak and 1885–2015 for ash. The oldest cambial age of oak is 285 years old, while the oldest ash is 130 years old. Two generations of oak were observed (young <100 years cambial age, old >100 years cambial age) and separated in further analysis (Supplementary Fig. 2c,d).

A high autocorrelation of raw series was found in old pedunculate oak (0.74) in comparison to ash (0.45), which points to the significant accumulated influence of climate conditions in the previous years. Comparison of standardized chronologies shows that narrow-leaved ash has higher mean sensitivity than old pedunculate oak (*MS*_*Ash*_ = 0.36 vs *MS*_*Oak_O*_ = 0.21). The interseries correlation (*Rbar*) is also higher for narrow-leaved ash (0.51) than for pedunculate oak (0.36). EPS value > 0.85 quantifies the degree to which the constructed chronology represents the hypothetical population (EPS_Ash_ from 1897, EPS_Oak_ from 1760).

### Climate-growth correlations

Simple linear correlation between residual chronologies of tree ring width index (TRWI) for ash and oak and monthly climate data showed that narrow-leaved ash was significantly more sensitive to the hydrologic component, especially precipitation, than pedunculate oak. A statistically positive correlation (*p*-value < 0.05) was determined for precipitation (Prec), river water level (R), river discharge (Q) and drought index (scPDSI) in individual monthly as well as individual seasonal values for 2- and 4-months intervals in the 1950–2014 period (Fig. 4).

The highest positive correlations were found for river discharge (*r* = 0.61) and water level (*r* = 0.52) in May of the current year as well as seasonal correlations from May to August (*r*_*Q*_ = 0.68, *r*_*R*_ = 0.52). Precipitation in April and July showed a significant positive correlation (*r* = 0.38). Highest correlation values were found from April to July (*r* = 0.63) for the seasonal values.

Significant positive correlations between the residual chronology and the drought index (scPDSI) for the individual month values were determined from February to September of the current year with the highest value in August (*r* = 0.61). Due to the high autocorrelation structure of scPDSI monthly values, a response function analysis that reduces individual month intercorrelation was performed. The results point to a significant positive sensitivity of ash to scPDSI values in July of the current year (Fig. 4). The seasonal values show the highest correlation from June to September (*r* = 0.60). Negative correlations for temperature were determined for individual months in May and June (*r* = −0.28) and for the seasonal values from May to June (*r* = −0.35).

Unlike narrow-leaved ash, radial growth in old pedunculate oaks showed significant sensitivity to scPDSI and temperature. Positive correlations with scPDSI were determined in July of the current year (*r* = 0.45). In the period from June to July, a peak in positive impact of scPDSI was recorded (*r* = 0.43). In contrast, a significant negative air temperature impact was determined for April (*r* = −0.35) and July (*r* = −0.39). The negative temperature impact becomes more pronounced looking at the seasonal values from April to July (*r* = −0.43).

Influence of the precipitation on radial growth is low but statistically significant. The highest value was determined in February of the current year (*r* = 0.26) and for seasonal values from February to May (*r* = 0.35). The same pattern was also observed for river discharge and water level. Both show the highest correlation values for individual months in May (*r*_*Q*_ = 0.31, *r*_*R*_ = 0.33) and for the seasonal values from April to July (*r*_*Q*_ = 0.35, *r*_*R*_ = 0.35)

Young oak trees showed lower climate sensitivity than old oaks, with the most significant positive correlation for temperature in June of previous year (*r* = 0.39) and for precipitation in June of current year (*r* = 0.27) (Fig. 4). We also found significant raw ring width decrease during drought events in the juvenile growth phase (Supplementary Fig. 5). Interestingly, in 2003 the driest year recorded did not affect the growth rate.

The results indicate significant difference in climate response between both species. Narrow-leaved ash exhibits a more stable signal for the studied period in comparison to oak. The correlation with the precipitation average for April–July showed a quite stable signal during the studied period. However, on the individual monthly level (May), significant increase in correlation with precipitation, river water level and river discharge were observed (Fig. 5a,c). Sensitivity to precipitation in April and May became more pronounced after 1950. A noticeable increase in response to the Sava River water level and river discharge in May was determined after the 1970s, and reciprocally there was an increase in the negative signal air temperature values in May (Fig. 5a).

Oak had a negative but not statistically significant signal for temperature until the 1950s, after which the signal becomes statistically significant, especially for April and July (Fig. 5b,d). At the same time, there were increases in the positive response to the precipitation, Sava River water level and river discharge in April, May and July. This pattern was also observed for the 1801–2014 period using HISTALP climate data. The negative temperature impact was present during the entire studied period, with an increasing trend that becomes especially pronounced after 1950 for all months between April and July as well as the seasonal average (April–July) (Fig. 5e).

### Historical evidence of disturbances and salvage logging data

According to the collected historical records for the period 1900–2010, 7.8 million m^3^ of dead oak trees have been salvaged. The first large dieback occurred from 1910–1925 (1.73 million m^3^) with three pronounced peaks in 1911, 1916 and 1924. From the 1950s until 1990s salvaging was low compared to the whole period. Still, two large individual events that were preceded by long-lasting floods (1965 and 1985) occurred in that period.

After the 1990s there is evidence of noticeable increase in salvage logging. During the last twenty years total salvage logging because of individual tree mortality was significantly higher than all the salvaged wood from the disturbance events put together. An increase in windthrow events has also been recorded (430153 m^3^ in the last two decades) as well as an increase in mortality for ash from 2010 onwards (Fig. 6).

The natural disturbance chronology analysis determined four pronounced periods of growth release in the pedunculate oak primeval forest (1780s, 1850s, 1890s and 1940s). Decadal peaks were determined for the following years: (I) 1788, (II) 1855, (III and IV) 1891 and 1893, and (V and VI) 1942, 1946 and 1950 (Fig. 7f). With narrow-leaved ash, three decades of growth release were determined (1940s, 1960s and 1980s), with peaks in 1946, 1950, 1966 and 1983 (Fig. 7h). These disturbance chronologies show a co-occurrence of growth release with summer droughts (Fig. 7a), high temperatures (Fig. 7b), low water level (Fig. 7c) and precipitation deficit (Fig. 7d) equally in both species. Co-occurrence of peaks in the chronology for both species was found during the 1940s when a series of dry years was recorded.

The successional response to natural disturbances in the form of cyclical regeneration was observed only once in the primeval oak forest, but not in ash stands. Oak age structure analysis showed one large period of recruitment that corresponded to the period of intense growth release in the 1940s (Fig. 7e). This age structure partially explains the sigmoidal shape of the diameter frequency distribution characteristic of primeval forests in the temperate zone. Apart from oak, the primeval forest reserve was found to have a significant distribution of common hornbeam with an average age of 45 years (20 cm in diameter). Its abundant occurrence coincides with the 1970s, that is, approximately 20 years after the recorded period of oak growth release.

## Discussion

Numerous studies in Europe suggest that high precipitation values and lower temperatures in the spring and summer increase the radial growth of pedunculate oak^34–42^.

The results of our research indicate that old oak trees are positively sensitive to scPDSI and hydrological parameters and at the same time limited by high air temperatures from April to July, whereas the role of precipitation is not as pronounced as that found in the above-mentioned research. Oak’s positive sensitivity to river water level & discharge in May could be attributed to more intensive new root hairs (<1 mm in diameter) growth during June and their abrupt dying in the surface layer of the soil during July and at the start of August^43,44^. Similar climate sensitivity was not observed for younger trees (<100 years), which we attribute to high interspecies competition (mainly with hornbeam).

In old oaks (older than 150 years) climate sensitivity is not stable over the analyzed period. The negative response to temperature (April–July) has become significantly pronounced in roughly the last seven decades (Figs 5 and 6), while at the same time the positive response to precipitation and hydrological parameters has been growing. This kind of change in oak’s response can be explained by extraordinary warm and drought years in the 1940s. (Fig. 2**)**. Similar temporal instability was observed in central-west Germany where oaks changed from a precipitation to temperature sensitivity after a severe drought in the 1940s^38^.

Unlike oak, ash exhibited a more pronounced sensitivity, especially to precipitation (April, May and July) and to river water level (May and August) while high temperatures in May and June reduced the radial growth. The aforementioned climatic signal in ash has been confirmed in rare researches in Europe^45,46^.

We attribute this pronounced precipitation signal to the extreme conditions of ash distribution in relation to oak. Ash occurs on heavy clay soil at the edge of alluvial swamps, which is also lower, concave terrain that retains rainfall longer and consequently increases the possibility of greater soil infiltration^47^. In such extreme conditions ash develops a specific shallow root system exposed to seasonal oscillations of extremely wet (spring, autumn) and dry periods (summer). Like other floodplain species, it is well adjusted to high levels of groundwater and limited by humidity shortage during drought periods^48^. Ash has a high tolerance for such extreme conditions, and it has no competition from any other tree species, which gives it the opportunity to dominate at the edges of wet lowland swamps^49,50^.

Climate sensitivity of ash is stable for seasonal correlations over the analyzed period but for monthly correlations (May) there is a constant increase of sensitivity to all variables especially hydrological parameters (Fig. 5a**)**. We explain this through increasing temperature and decreasing in moisture (decrease in river water level - declining trend −2.24 cm*year^−1^ in May for the 1926–2014 period).

The natural dynamics and structure of lowland floodplain primeval forests is hard to reconstruct due to the lack of preserved reserves in Europe, most of them are deforested during the end of the 19^th^ century (Supplementary Fig. 6). According to historical data four severe individual events were evident during the past century (Fig. 6). Such events occurred on 5.5% of the whole lowland area. Intensity of this recorded events was from 46–139 m^3^ ha^−1^ (mean 70 m^3^ ha^−1^). The most severe dieback was in 1985 with intensity of 139 m^3^ ha^−1^. The main cause for such events was a combination of drought follow by floods of long duration. We also found an increase of windthrow events especially during last past decades which can be attributed to the difference in resistance to natural disturbances of managed and semi-natural forests in contrast to primeval ones^32^. In our research three pronounced periods of growth release correspond to droughts. Age structure of these forests shows only two generations of oak trees revealing the severe drought of 1940s as the only drought that triggered a regeneration. Origin of first generation of oak also corresponds to drought during the 1740s (Fig. 7).

This implies the complexity of natural regeneration dynamic in primeval forests and also the significant role of drought in development of uniformed age structure of European lowland primeval forests. The lack of regeneration after the disturbances in the 1850s and 1890s (Fig. 7e**)** can be attributed to the fact that oak regenerates in a small area, is especially dependent on light conditions and is spatially limited by the heavy seed dispersion mechanism. Another possible factor is high competition with field elm (*Ulmus minor* L.) which has been widely distributed until 1970s in floodplain forests. Also unavoidable is the possible influence of herbivores (primarily wild pigs) and birds, which can significantly decrease abundant seed crops^51^. Observed growth release of ash trees did not result in regeneration. Being a pioneer tree species, ash originated through succession on the wetlands and the structure of these forests are very dense which hinders successful regeneration. Therefore, it requires large scale disturbances for adequate regeneration in contrast to oak.

Rising temperatures and anthropogenic influence have significantly changed the hydrologic conditions in both the river basin area and the river itself^52^. This study, which was conducted in the still preserved natural retention area of the Sava River, also found significant changes. They manifest in the decline of average, and especially minimal annual and individual monthly water levels, with particular intensity after 1980 (Fig. 2). Such negative trends can be explained by increased evaporation due to increases in temperature and evapotranspiration and have been recorded in most European rivers^52^. From an ecological standpoint, river water level is an important indicator of groundwater levels that recharges by infiltration from the Sava riverbed during high water levels (spring and autumn) even up to 5 km from the riverbed^53^.

The proportion of transpiration in floodplain forests is a high 80% of the total evapotranspiration, and most of the transpired water originates from underground sources^54^. In such conditions, climate change together with the fall of river water levels, increasingly expose oak to unfavorable conditions^37,55–58^.

The results of this research confirm recent trends that higher air temperatures hasten the long-term decline of trees and that in the dry conditions of this area, oak might be greatly endangered by changes in climate^59–63^. Although some research suggests an increase in oak radial growth due to higher temperatures^56,64^, CO_2_ fertilization and increases in N deposition^65^, we believe this might be possible only in normal hydrologic conditions.

## Conclusions

We conclude that pedunculate oak and narrow-leaved ash differ in their sensitivity to climate and hydrological parameters to which ash is more sensitive. Constant increase of sensitivity to precipitation and river water level of ash has become more pronounced during the climate warming period. Our results suggest that extreme climatic events, especially drought are a significant driver behind growth release but isn’t always followed by successful recruitment in the studied forests. We also found that oak has an evident shift in sensitivity triggered by severe droughts in the 1940s. Such shift may have been due to its adaptation strategy to increasing temperature and drier environment conditions.

Narrow-leaved ash, as the colonizer of wet swamp edges, could also face the rising pressure of water deficit. Its climate signal is slightly more stable, without the dramatic changes exhibited by oak, but considering the disturbance dynamic reconstruction results, it shows greater sensitivity to droughts.

The physiological flexibility of oak’s adaptation to drought events was shown to be an optimal survival mechanism in the past. However, it could be expected that in disturbed conditions with declining levels of groundwater, oak might not be able to utilize this alternative in the future.

## Electronic supplementary material

Supplementary Information
