# Supplementary material for: Drought-induced shift in tree response to climate in floodplain forests of Southeastern Europe
Source: Sci Rep. 2018 Nov 7;8:16495. doi: 10.1038/s41598-018-34875-w (PMC6220280; doi:10.1038/s41598-018-34875-w)
Supplement: Supplementary file 1 — Supplementary Information [file 41598_2018_34875_MOESM1_ESM.docx]

**Drought-induced shift in tree response to climate in floodplain forests of Southeastern Europe**

Stjepan Mikac^*1, 2^, Anja Žmegač^1, 2^, Domagoj Trlin^1^, Vinko Paulić^1^, Milan Oršanić^1^, Igor Anić^1, 3^

**Appendix S1: supplemental figures and tables**

**Table 1.** Descriptive statistics of analyzed raw and standardized tree-ring width series.

**Figure 1.** Tree-ring cores and wood sample collection in Special forest reserve Prašnik from live trees and from large wood debris

**Figure 2.** Raw, tree-ring widths series dated in site chronologies

**Figure 3.** Comparison of residual chronologies used different standardization methods.

**Figure 4.** Results of the bootstrap correlation analysis between tree rings residual chronologies

**Figure 5.** Drought years induce growth decrease in younger phase of oak trees growth.

**Figure 6.** Historical images from end of the 19 century.

**Supplementary Table 1.**  Descriptive statistics of analyzed raw and standardized tree-ring width series.

| **Samples** | **Trees (Cores)** | **Span** | **EPS > 0.85** | **Rbt** | **Rwt** | **Rbar** | **MS** | **Mean ± SD** | **GLK** | **AC1** |
| --- | --- | --- | --- | --- | --- | --- | --- | --- | --- | --- |
| Raw ring width series | | | | | | | | | | |
| *Oak_old* | 80 (138) | 1732-2017 | 1765 | 0.36 | 0.69 | 0.36 | 0.21 | 2.08+-0.90 | 0.63 | 0.74 |
| *Oak_young* | 45 (77) | 1908 - 2017 | 1927 | 0.52 | 0.75 | 0.54 | 0.24 | 2.48+1.05 | 0.62 | 0.71 |
| *Ash* | 47(85) | 1885-2015 | 1899 | 0.43 | 0.69 | 0.44 | 0.36 | 2.60+-1.27 | 0.69 | 0.45 |
| Standardized series using spline method | | | | | | | | | | |
| *Oak_old* | 80 (138) | 1732-2017 | 1760 | 0.36 | 0.68 | 0.36 | 0.21 | 1.00+-0.30 | 0.63 | 0.54 |
| *Oak_young* | 45 (77) | 1908 - 2017 | 1949 | 0.34 | 0.66 | 0.35 | 0.24 | 1.00+0.29 | 0.63 | 0.41 |
| *Ash* | 47(85) | 1885-2015 | 1897 | 0.50 | 0.73 | 0.51 | 0.36 | 1.00+-0.42 | 0.69 | 0.30 |

Number of analysed samples (Trees(Cores)), Chronology Time span (Span), Period of Expressed population signal >0.85 (EPS > 0.85), Mean correlation between trees (Rbt), Mean correlation within-trees (Rwt), Mean correlation among all series (Rbar), Mean Sensitivity (MS), Mean and standard deviation of ring width (Mean ± SD), Gleichläufigkeit index (GLK- a measure of the year-to-year agreement between samples) and first-order autocorrelation (AC1).


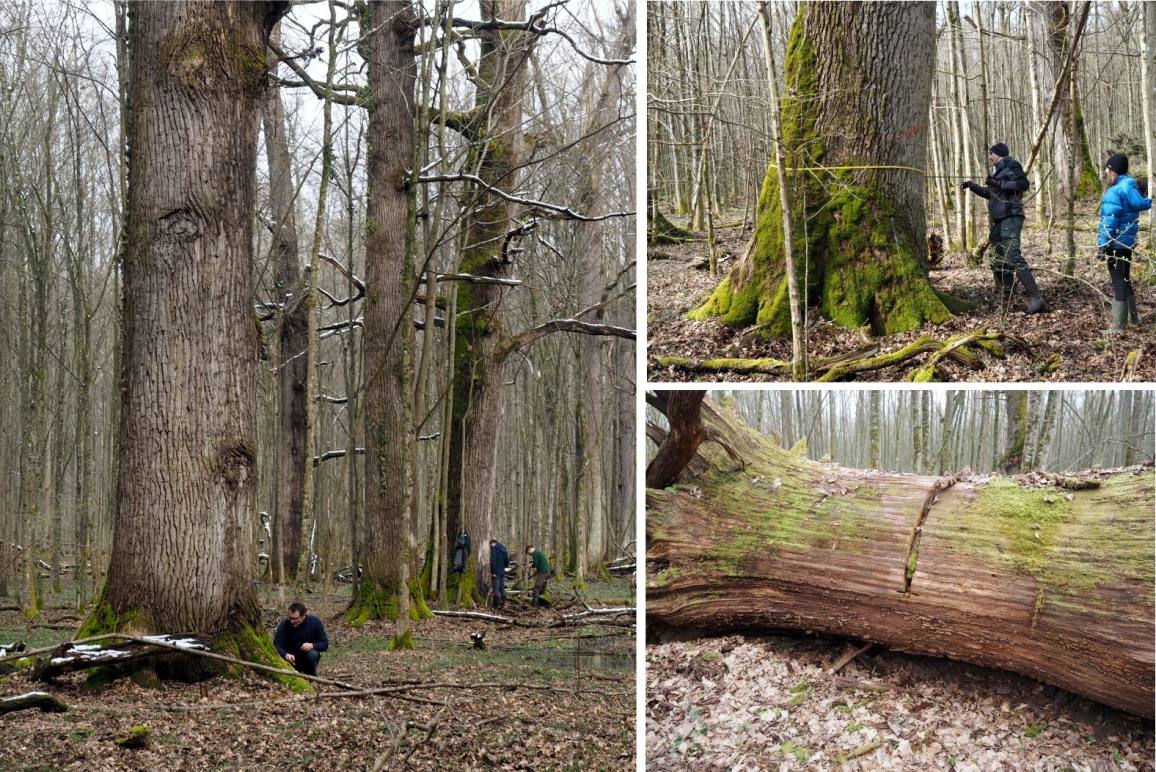


**Supplementary Figure 1.**  Tree-ring cores and wood sample collection in Special forest reserve Prašnik from live trees and from large wood debris.


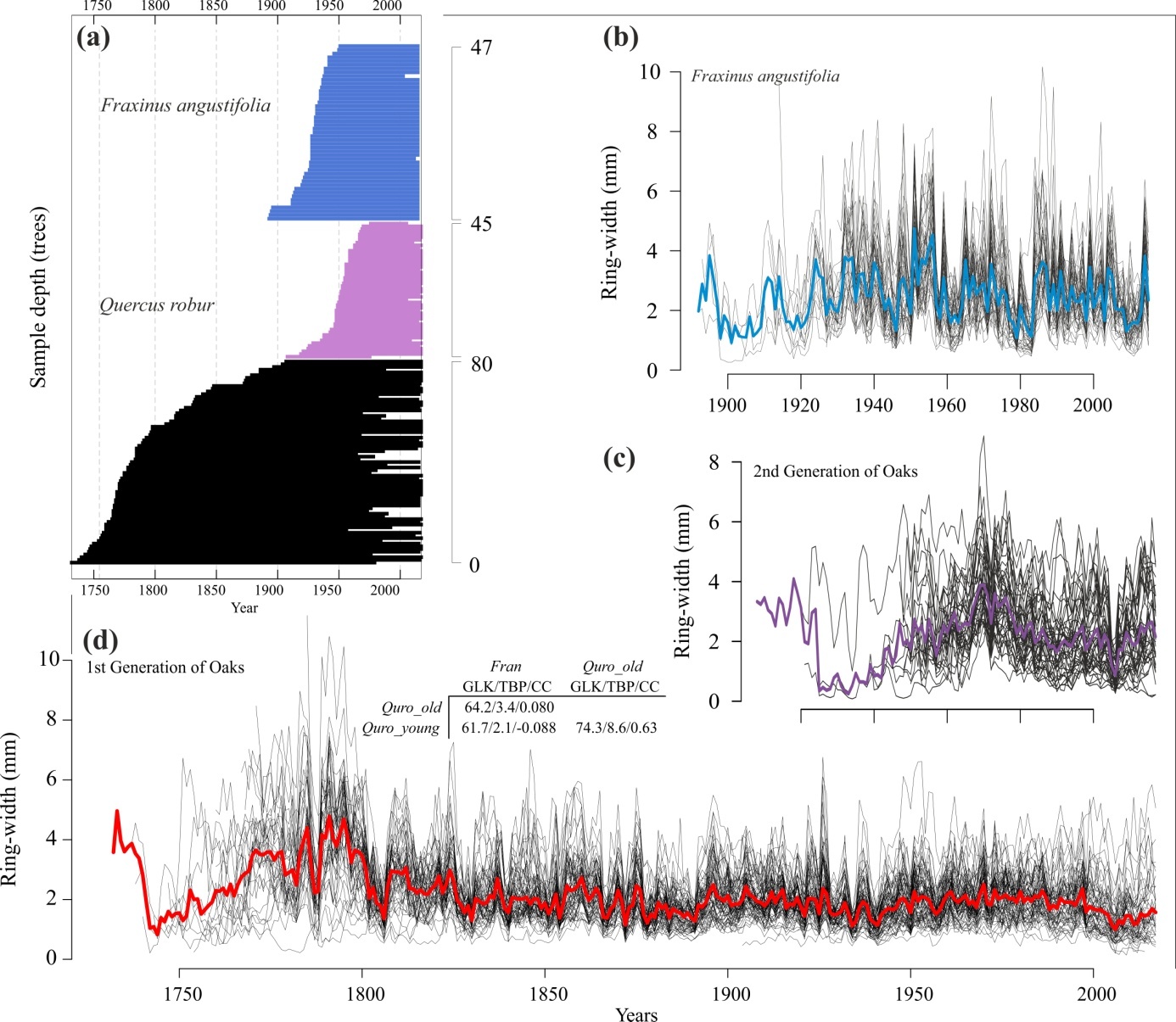


**Supplementary Figure 2.**  Raw, tree-ring widths series dated in site chronologies, narrow-leaved ash (b), second generation of oaks (c) and first generation of oaks (d). Sample depth of average series per trees for ash and oaks (a). Bolded line showed average values of ring width data. Table represent mean chronologies comparison where are: GLK, TBP and CC.

**
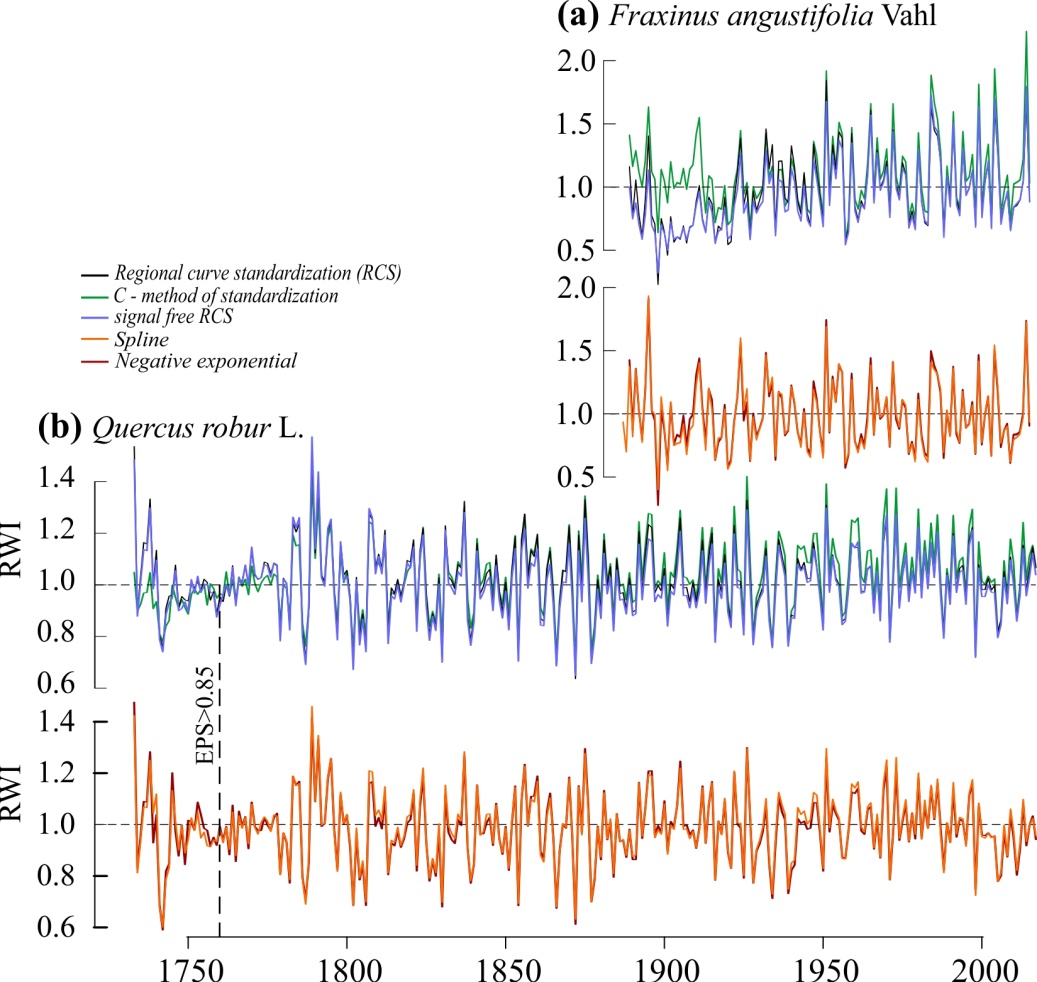
**

**Supplementary Figure 3.** Comparison of residual chronologies used different standardization methods.

**
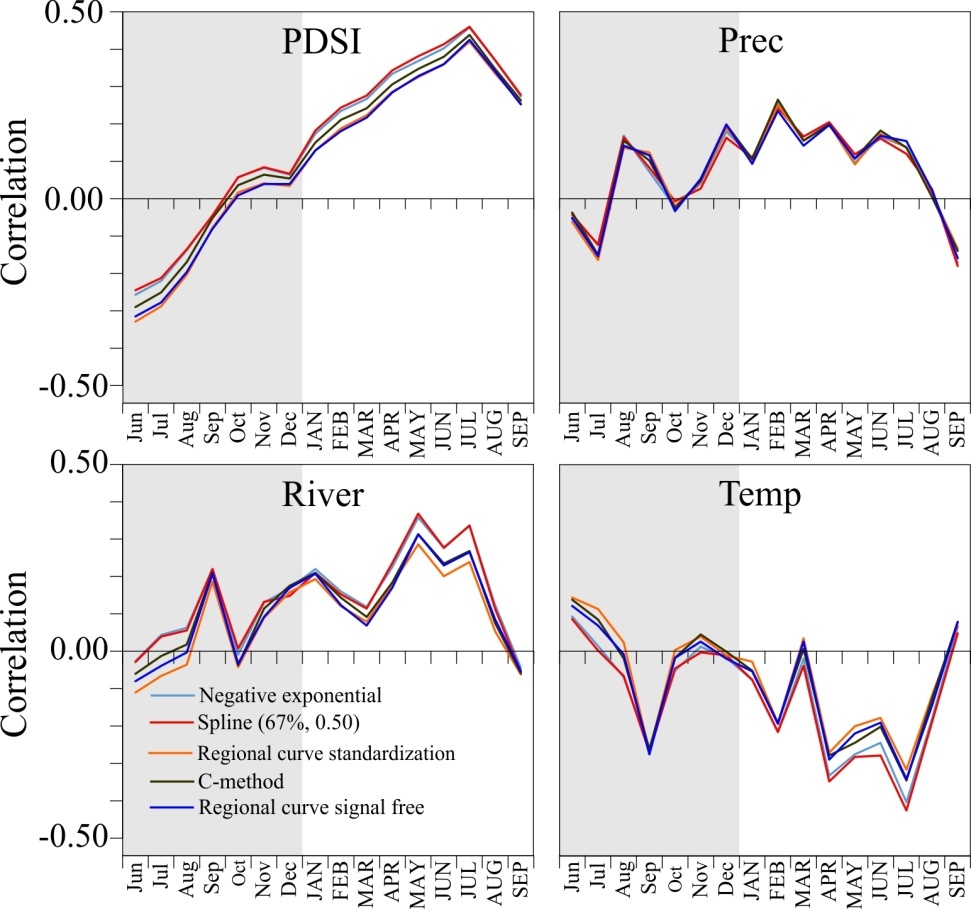
**

**Supplementary Figure 4.**  Results of the bootstrap correlation analysis between tree rings residual chronologies (TRWI) produced by different methods of standardization and monthly climate factors (PDSI, Precipitation and mean monthly temperature) and mean monthly Sava River water level (River).


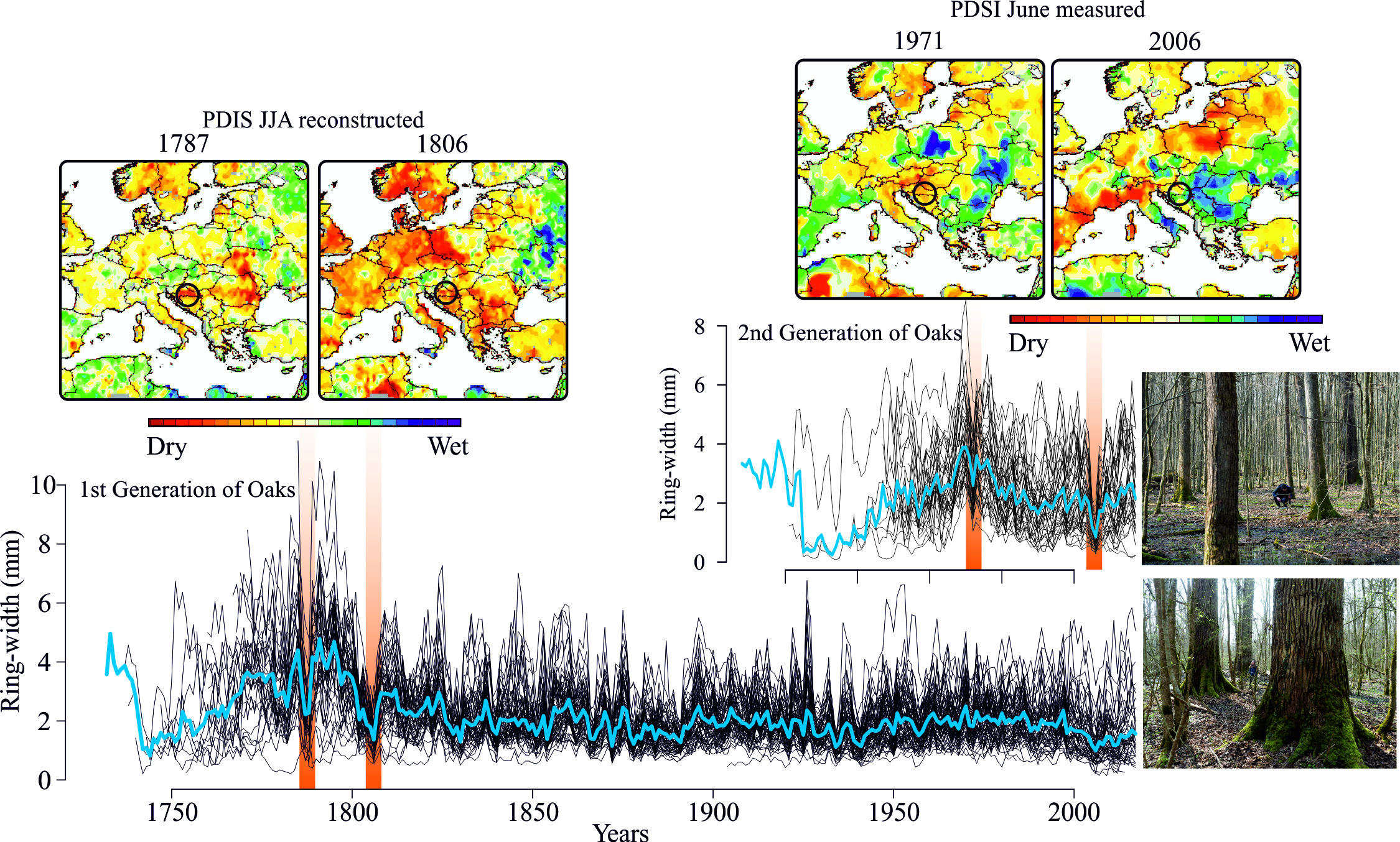


**Supplementary Figure 5.**  Drought years induce growth decrease in younger phase of oak trees growth.


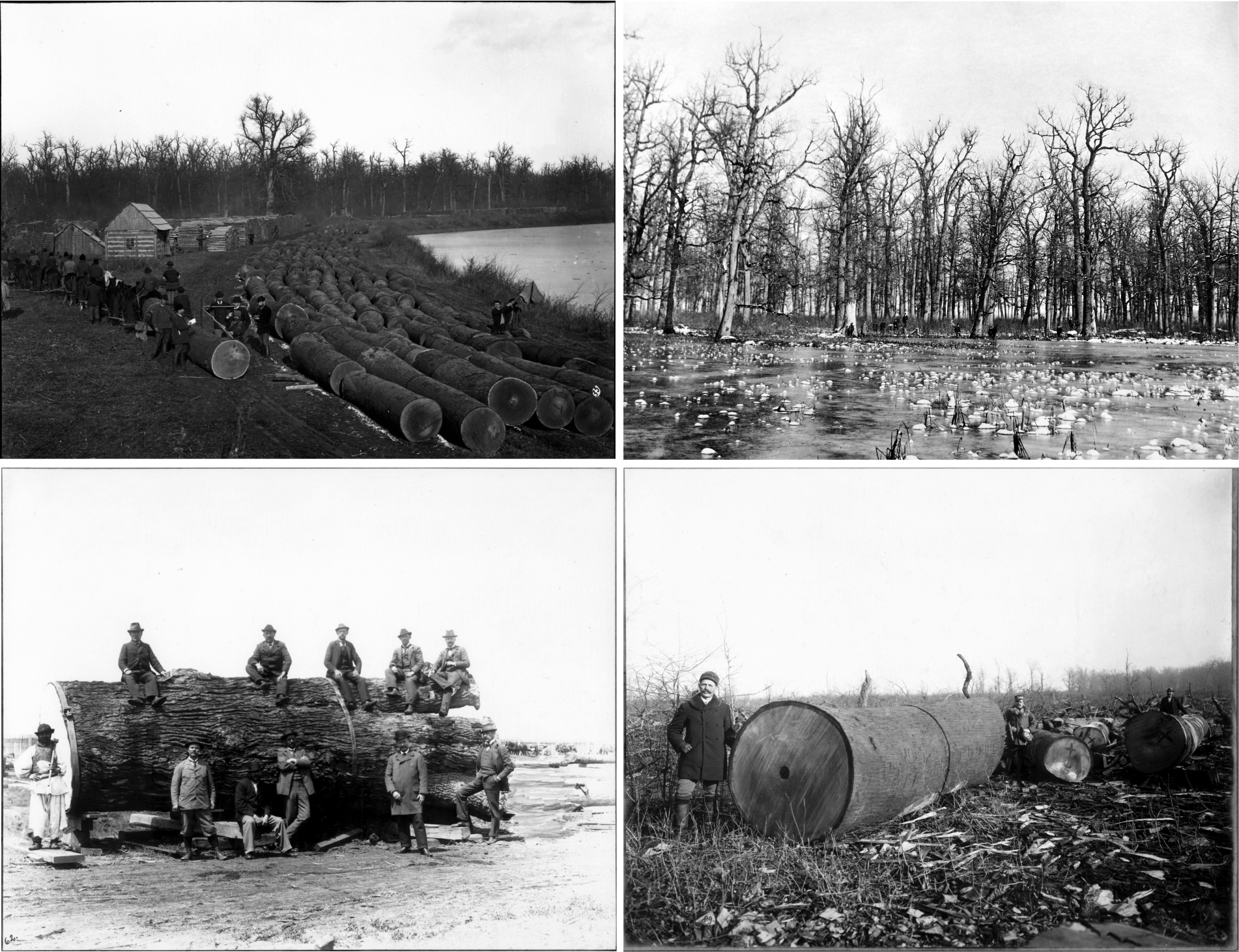


**Supplementary Figure 6.**  Historical images from end of the 19^th^ century. Deforestation of primeval floodplain forests in Croatia.
